# Use of serum osmolality to identify heart disease stage in dogs and relationship to mathematical chloride correction

**DOI:** 10.1111/jvim.16863

**Published:** 2023-09-13

**Authors:** Edward J. Daly, Autumn N. Harris, Darcy B. Adin

**Affiliations:** ^1^ Department of Small Animal Clinical Sciences College of Veterinary Medicine, University of Florida Gainesville Florida USA

**Keywords:** antidiuretic hormone, cardiovascular, chloride, diuretic resistance, heart failure

## Abstract

**Background:**

Heart failure‐associated hypochloremia can be depletional from diuretics or dilutional from water retention. Serum osmolality reflects water balance but has not been evaluated in dogs with heart disease.

**Hypothesis:**

To determine if serum osmolality is related to heart disease stage and amount of mathematical correction of serum chloride (Cl^−^) concentrations in healthy dogs and dogs with myxomatous mitral valve degeneration (MMVD).

**Animals:**

Seventy‐seven dogs (20 healthy, 25 Stage B MMVD, 32 Stage C/D MMVD).

**Methods:**

Serum Cl^−^ concentrations were mathematically corrected. Osmolality was calculated (calOsm) and directly measured by freezing point depression (dmOsm) and compared by Bland‐Altman analysis. Biochemical variables and osmolality were compared among healthy, Stage B, and Stage C/D dogs. Correlations were explored between osmolality and biochemical variables. Median and range are presented. *P* < .05 was considered significant.

**Results:**

The calOsm was different among groups (*P* = .003), with Stage B (310 mOsm/kg; 306, 316) and C/D dogs (312 mOsm/kg; 308, 319) having higher calOsm than healthy dogs (305 mOsm/kg; 302, 308). Osmolality methods were moderately correlated (*P* < .0001, *r*_s_ = .46) but with proportional bias and poor agreement. The amount of Cl^−^ correction was negatively correlated with calOsm (*P* < .0001, *r*_s_ = −.78) and dmOsm (*P* = .004, *r*_s_ = −.33). Serum bicarbonate concentration was negatively correlated with Cl^−^ (*P* < .0001, *r*_s_ = −.67).

**Conclusions and Clinical Importance:**

Dogs with Stage B and Stage C/D heart disease had higher calOsm than healthy dogs. Osmolality was inversely related to the amount of Cl^−^ correction, which supports its use in assessing relative body water content. Poor agreement between calOsm and dmOsm prevents methodological interchange.

**Abbreviations**
ACVIM: American College of Veterinary Internal Medicine
ADH: antidiuretic hormone
BUN: blood urea nitrogen
calOsm: calculated serum osmolality
cCl^−^
: corrected serum chloride concentration
CHF: congestive heart failure
Cl^−^
: chloride
dmOsm: directly measured serum osmolality
IV: intravenous
mCl^−^
: measured serum chloride concentration
MMVD: myxomatous mitral valve disease
Na^+^
: sodium
PO^4−^
: phosphorus

## 1 INTRODUCTION

Congestive heart failure (CHF) in dogs is a clinical syndrome that can result from long‐standing structural disorders of the heart, such as myxomatous mitral valve disease (MMVD) and dilated cardiomyopathy.^1^ This syndrome is characterized by decreased cardiac output and inadequate tissue perfusion, leading to increased filling pressures and congestion. To compensate for the impaired perfusion, activation of the renin‐angiotensin‐aldosterone system mediates retention of sodium and volume expansion while osmolality is maintained. However, advanced CHF creates a volumetric stimulus for release of antidiuretic hormone (ADH), dissociating release of the hormone from osmoregulation and creating hyponatremia and hypochloremia, both of which have been associated with poor prognosis.^2^, ^3^, ^4^, ^5^, ^6^ Hypochloremia is further exacerbated by urinary chloride (Cl^−^) loss caused by loop diuretic inhibition of the Na^+^/K^+^/2Cl^−^ cotransporter in the ascending loop of Henle.^4^, ^7^

Non‐osmotic ADH release is suspected to contribute to the pathogenesis of refractory CHF with poor diuretic response (known as Stage D). Still, the clinical distinction between dogs with Stage C (controlled CHF) and Stage D is challenging. Current guidelines use only the diuretic dosage needed for CHF control to identify Stage D dogs, but this approach has not been validated.^1^ We previously showed that hypochloremia and upward mathematical Cl^−^ correction differentiate Stage C from Stage D CHF.^4^ Excessive water retention is a potential cause of hypochloremia, as evidenced by upward mathematical correction of serum Cl^−^, but this association has not been proven. If true, excessive water retention through ADH influence is a possible mechanism whereby diuretic resistance might occur in Stage D dogs. Measurement of body water content in the whole animal is challenging, and although an enzyme immunoassay has been validated for the measurement of ADH in dogs, this type of testing is not easily carried out in the clinical setting.^8^ Serum osmolality, however, is a simple measure of the concentration of solutes in serum, providing information on water content relative to electrolyte content. Investigation into the role of serum osmolality to identify refractory CHF is a first step toward targeting treatment to the specific cause of poor diuretic response, and might provide additional information to improve heart disease staging.

Our objectives were to determine if serum osmolality is related to heart disease stage and the degree of mathematical correction of serum Cl^−^. We hypothesized that dogs with CHF (Stages C and D) would have lower serum osmolality than healthy dogs and dogs with preclinical heart disease (Stage B), and that dogs with Stage D CHF would have lower serum osmolality than dogs with Stage C CHF. Additionally, we hypothesized that serum osmolality would be indirectly correlated with the degree of mathematical Cl^−^ correction.

## 2 MATERIALS AND METHODS

Ours was a prospective, observational study of dogs with MMVD that were presented to the cardiology service or the primary care service at the University of Florida Small Animal Hospital. Informed client consent was obtained from all owners. The Institutional Animal Care and Use Committee at the University of Florida Small Animal Hospital approved blood collection for this study (202120518).

### 2.1 Animals

Dogs with heart disease secondary to MMVD and healthy control dogs were included in the study. Dogs were enrolled if their owners provided consent for blood collection or use of residual serum samples after clinically indicated blood tests. Exclusion criteria were heart disease other than MMVD (eg, dilated cardiomyopathy, congenital heart disease) and clinically relevant extracardiac disease (eg, gastrointestinal disease, diabetes mellitus). Dogs were separated into 3 groups based on the presence or absence of, and severity of heart disease using criteria established by the American College of Veterinary Internal Medicine (ACVIM) Guidelines.^1^ Healthy control dogs did not have clinical or physical examination evidence of heart disease and were primary care clinic patients. Dogs with heart disease were cardiology service patients and categorized as Stage B (preclinical, no CHF) or Stage C and D (controlled or refractory CHF). Dogs were identified as having Stage B1 (no evidence of cardiac enlargement) or B2 (cardiac enlargement present) based on results of echocardiography and radiography, but were considered a single group during analysis. Stage C and D dogs were considered together as a group because of the small number of Stage D dogs. However, results for Stage D dogs were marked graphically for viewing of these results. Acute or chronic CHF status was noted for Stage C/D dogs based on whether they had evidence of systemic congestion at the time of sampling (eg, dyspnea caused by pulmonary edema, ascites). The amount of IV furosemide administered before blood sampling was recorded for dogs with acute CHF. Only 1 sample per dog was included in the study. Clinical variables recorded for each dog included signalment, body weight, sex, ACVIM heart disease stage, presence or absence of acute CHF, medications, and dosages.

### 2.2 Serum renal panel

One milliliter of whole blood was obtained by peripheral venipuncture, placed in a non‐additive tube, and centrifuged for 4 minutes at 8000 rpm after clot formation to obtain at least 250 μL of serum. Serum renal panels were performed using an AU480 Chemistry Analyzer (Beckman Coulter, Brea, CA) in the Clinical Pathology Laboratory at the University of Florida. This chemistry analyzer is calibrated daily to ensure all measured results are within 3 SD above and below the mean. Renal panel variables included glucose, albumin, blood urea nitrogen (BUN), creatinine, sodium (Na^+^), potassium, Cl^−^, bicarbonate (HCO_3_^−^), calcium, phosphorus (PO_4_^−^), and anion gap. The laboratory‐reported calculated serum osmolality (calOsm) also was included on the panel using the BUN, Na^+^, and glucose results (calOsm = 2[Na^+^] + [glucose/18] + [BUN/2.8]).^9^ Although there is a subtle distinction between osmolality and osmolarity, the differences are clinically unimportant and therefore osmolality is used when reporting the calculated or directly measured results.^9^, ^10^, ^11^ The measured serum chloride concentration (mCl^−^) was mathematically corrected to obtain the corrected serum chloride concentration (cCl^−^) using the following equation^4^: (mid‐reference range Na^+^/measured Na^+^) × mCl^−^. The amount of chloride correction was determined by subtracting the mCl^−^ from the cCl^−^.

### 2.3 Direct osmolality measurement

Residual serum samples were collected after the measurement of renal panel variables by the Clinical Pathology Laboratory. The presence or absence of hemolysis or lipemia was noted in samples. Following guidelines used in previous studies, samples were stored at 4°C (refrigeration) when measured the same day or stored at −18°C (freezer) for up to 5 days before retrieval for direct osmolality measurement.^10^, ^12^ Before measurement, frozen samples were thawed at room temperature for approximately 15 minutes before direct measurement of serum osmolality (dmOsm) by freezing point depression,^13^ utilizing a 5004 MICRO‐OSMETTE Automatic High Sensitivity 50 μL Osmometer (Precision Systems, Natick, Massachusetts). Immediately before measuring multiple samples, the osmometer was calibrated 3 times utilizing 50 μL of a commercially available osmometry standard of 500 mOsm/kg H_2_O control solution. One calibration of a 50 μL standard 100 mOsm/kg H_2_O control solution then was measured to ensure the osmometer accurately detected unknown samples. All serum osmolality measurements were directly determined by the osmometer using a minimum of 50 μL serum and reported as mOsm/kg H_2_O. All osmometer calibrations and measurements were run in Precision Systems osmomette specialty tubes. The osmole gap was calculated by subtracting calOsm from dmOsm.

### 2.4 Statistical analysis

The data were compiled in an Excel spreadsheet and statistically analyzed using commercially available software (Graph Pad Prism version 9). Descriptive and clinicopathologic data for dogs in each ACVIM heart disease stage were tested for normality using the Kolmogorov‐Smirnov test and presented as the median and range (minimum, maximum). Clinicopathologic variables (including dmOsm and calOsm) were compared among healthy dogs, Stage B dogs, and Stage C/D dogs using Kruskal‐Wallis test or 1‐way ANOVA, depending on normality testing, with Dunn's test or Holm‐Šídák's test, respectively, performed for multiple comparison testing. An unpaired *t*‐test or Mann‐Whitney test was used to compare variables between Stage B1 and B2 dogs and between Stage C/D dogs with acute and chronic CHF, whereas the Chi‐squared test for trends or Fisher's exact test was used to compare sex and heart disease stages for these comparisons. A subset of samples (22 previously refrigerated and 29 previously frozen) was used to evaluate for effects of freezing, hemolysis, and lipemia on dsOsm using multiple linear regression. Hemolysis and lipemia were considered either present or absent for statistical purposes. The correlation between osmolality and renal panel variables was explored for all enrolled dogs using Spearman's correlation test. Measurement agreement between calOsm and dmOsm was assessed using Bland‐Altman analysis, including all dog samples. *P* < .05 was considered significant, except for biochemical comparisons where Bonferroni's correction was applied and indicated an adjusted *P* ≤ .003 was appropriate to minimize the risk of false positive results. Outliers were not removed because of the potential for biologic variability in the absence of a non‐biological explanation.

## 3 RESULTS

### 3.1 Animals

Twenty healthy dogs, 25 Stage B dogs (10 B1, 15 B2), and 32 Stage C/D dogs (26 C, 6 D) were enrolled between May 16, 2022, and December 22, 2022. There were no significant differences in demographic or clinicopathologic data between B1 (n = 10) and B2 (n = 15) dogs (*P* > .09 all; Table S1), justifying their consideration as a single group of preclinical MMVD dogs. Because only 6 Stage D dogs were enrolled, this group was not analyzed separately, and the 32 Stage C and Stage D dogs were combined for analysis. Individual data points for the 6 Stage D dogs are highlighted on the graphs.

Healthy dog breeds included mixed breed (n = 9) and 1 each of Shih Tzu, Beagle, Bulldog, Karelian Bear Dog, Vizla, Newfoundland, Dalmatian, Labrador Retriever, Italian Greyhound, Doberman Pinscher, and American Pit Bull Terrier. Stage B dog breeds included mixed breed (n = 7), Cavalier King Charles Spaniel (n = 5), Chihuahua (n = 4), Dachshund (n = 3), Maltese (n = 2), and 1 each of American Pit Bull Terrier, Japanese Spitz, Shetland Sheepdog, and Jack Russell Terrier. Stage C/D dog breeds included mixed breed (n = 6), Shih Tzu (n = 6), Chihuahua (n = 5), Boston Terrier (n = 3), Maltese (n = 2), Miniature Schnauzer (n = 2), Cavalier King Charles Spaniel (n = 2), and 1 each of Havanese, Chinese Crested, Pekingese, Jack Russell Terrier, English Toy Spaniel, and American Cocker Spaniel.

Dosages of common cardiac medications for dogs in each group are shown in Table 1. Noncardiac medications for healthy dogs included gabapentin (n = 2) and 1 each of meloxicam, trazadone, allopurinol, and carprofen. Noncardiac medications for Stage B dogs included amlodipine (n = 5), gabapentin (n = 3), levetiracetam (n = 3), grapiprant (n = 2), sildenafil (n = 2), and 1 each of VetriFlex, CBD oil, skin/joint supplement, probiotic, hydrocodone, L‐theanine, doxycycline, amoxicillin‐clavulanic acid, piroxicam, selegiline, capromorelin, metronidazole, trazodone, levothyroxine, lactulose, polyethylene glycol 2250, psyllium fiber, taurine, polysulfated glycosaminoglycan, phenobarbital, and S‐adenosylmethionine. Noncardiac medications for Stage C/D dogs included hydrocodone (n = 7), amlodipine (n = 6), sildenafil (n = 3), and 1 each of inhaled budesonide, trazodone, clopidogrel, fluoxetine, potassium gluconate, theophylline, and propafenone.

**TABLE 1:** Age, weight, medication dosages, serum biochemical variables, and osmolality for healthy dogs, Stage B dogs and Stage C/D dogs were compared using Kruskal‐Wallis test or one‐way ANOVA, depending on normality testing, with Dunn's test or Holm‐Šídák's test, respectively, performed for multiple comparison testing.

Variable	Healthy (n = 20)	Preclinical Stage B1/B2 (n = 25)	CHF Stage C/D (n = 32)	*P* value
Sex	1 MI, 15 MN, 0 F, 4 FS	2 MI, 11 MN, 0 FI, 12 FS	0 MI, 17 MC, 2 FI, 13 FS	.14
Age (months)	100 (39, 138)	139 (57, 190)^a^	132 (86, 171)^a^	**<.0001**
Weight (kg)	26.3 (7.5, 63.0)	6.4 (2.4, 30.7)^a^	5.6 (2.0, 13.9)^a^	**<.0001**
Pimobendan mg/kg/day	n = 0	0.62 (0.47, 0.96) (n = 15)	0.75 (0.36, 1.67) (n = 29)	.02
IV furosemide if hospitalized	n = 0	n = 0	11.1 (2.0, 24.5) (n = 10)	ND
Furosemide mg/kg/day	n = 0	1.7 (n = 1)	4.0 (0.0, 13.4) (n = 25)	ND
Torsemide mg/kg/day	n = 0	n = 0	0.43 (0.26, 1.01) (n = 5)	ND
ACE‐I mg/kg/day	n = 0	0.86 (0.38, 1.45) (n = 5)	0.63 (0.28, 1.01) (n = 23)	.39
Spironolactone mg/kg/day	n = 0	3.51 (3.16, 3.85) (n = 2)	2.22 (0.47, 3.44) (n = 21)	.02
Glucose mg/dL	101 (75, 122)	94 (43, 122)	100 (62, 141)	.12
Albumin mg/dL	3.27 (2.26, 3.53)	3.10 (2.22, 4.16)	3.42 (2.66, 4.23)	.06
BUN mg/dL	16 (10, 40)	24 (13, 70)^a^	33 (13, 114)^a^	**<.0001**
Creatinine mg/dL	1.03 (0.77, 1.44)	0.92 (0.55, 1.99)	1.12 (0.44, 3.80)	.15
Na^+^ mEq/L	146.9 (140.9, 151.3)	148.0 (144.2, 153.6)	147.8 (141.0, 152.8)	.12
K^+^ mEq/L	4.4 (3.7, 5.0)	4.5 (3.8, 5.5)	4.4 (3.3, 5.1)	.1
Cl^−^ mEq/L	111.5 (104.7, 117.9)	110.5 (106.6, 123.6)	105.3 (93.6, 112.9)^a^ ^,^ ^b^	**<.0001**
cCl^−^ mEq/L	111.7 (108.7, 114.0)	110.0 (106.1, 119.2)	103.6 (95.8, 109.6)^a^ ^,^ ^b^	**<.0001**
Amount of Cl^−^ correction mEq/L	−0.45 (−3.94, 3.98)	−1.35 (−5.91, 1.58)	−1.11 (−4.84, 3.52)	.11
HCO_3_^−^ mEq/L	20 (18, 25)	21 (13, 26)	24 (17, 29)^a^ ^,^ ^b^	**<.0001**
Ca^+2^ mg/dL	10.0 (8.8, 11.1)	10.2 (9.4, 11.3)	10.7 (9.3, 12.6)^a^	**.002**
PO4^−^ mg/dL	3.4 (1.8, 4.5)	3.5 (2.3, 5.3)	4.2 (2.1, 7.9)^a^ ^,^ ^b^	**.001**
Anion gap	18.8 (15.6, 21.2)	20.3 (14.8, 25.8)	22.35 (18.0, 31.0)^a^ ^,^ ^b^	**<.0001**
calOsm	305.4 (291.7, 315.8)	310.0 (299.7, 328.2)^a^	312.2 (293.5, 342.9)^a^	**.003**
dmOsm	291.5 (279.0, 345.0)	296.0 (279.0, 498.0)	297.5 (247.0, 388.0)	.48
Osmole gap	−10.6 (−23.6, 42.3)	−15.1 (−27.2, 174.6)	−17.3 (−64.7, 77.5)	.34

*Note*: Median (minimum, maximum) values for each variable are shown. The Chi‐square test was used to compare sex between groups. Significant differences at the adjusted *P* ≤ .003 are bolded.

Abbreviations: ACE‐I, angiotensin‐converting enzyme inhibitor; BUN, blood urea nitrogen; Ca^+2^, calcium; calOsm, calculated osmolality; cCl^−^, corrected chloride; Cl^−^, chloride; dmOsm, direct measured osmolality; FI, female intact; FS, female spayed; HCO_3_^−^, bicarbonate; K^+^, potassium; MI, male intact; MN, male neutered; Na^+^, sodium; ND, not done; PO_4_^−^, phosphorus.

^a^ Indicates significantly different compared with healthy dogs.

^b^ Indicates significantly different compared with Stage B dogs.

### 3.2 Effects of sample preparation and qualities

Multiple linear regression did not show an effect of prior freezing (*P* = .82), hemolysis (*P* = .28), or lipemia (*P* = .89) on dsOsm.

### 3.3 Group differences (Table 1)

Healthy dogs were younger and heavier than dogs with Stage B or C/D MMVD, but there were no demographic differences between Stage B and Stage C/D dogs. Serum biochemical differences among groups for HCO_3_^−^, PO_4_^−^, and anion gap were characterized by higher results for Stage C/D dogs compared with both healthy dogs and Stage B dogs (*P* ≤ .001), with no differences between healthy dogs and Stage B dogs. Stage B and Stage C/D dogs had higher BUN than healthy dogs (*P* < .0001), but there was no difference between Stage B and Stage C/D dogs. Serum creatinine concentration was not different among groups (*P* = .15). Serum Cl^−^ and cCl^−^ were lower for Stage C/D dogs compared with both healthy dogs and Stage B dogs (*P* < .0001) but there were no differences between healthy dogs and Stage B dogs.

**FIGURE 1:**
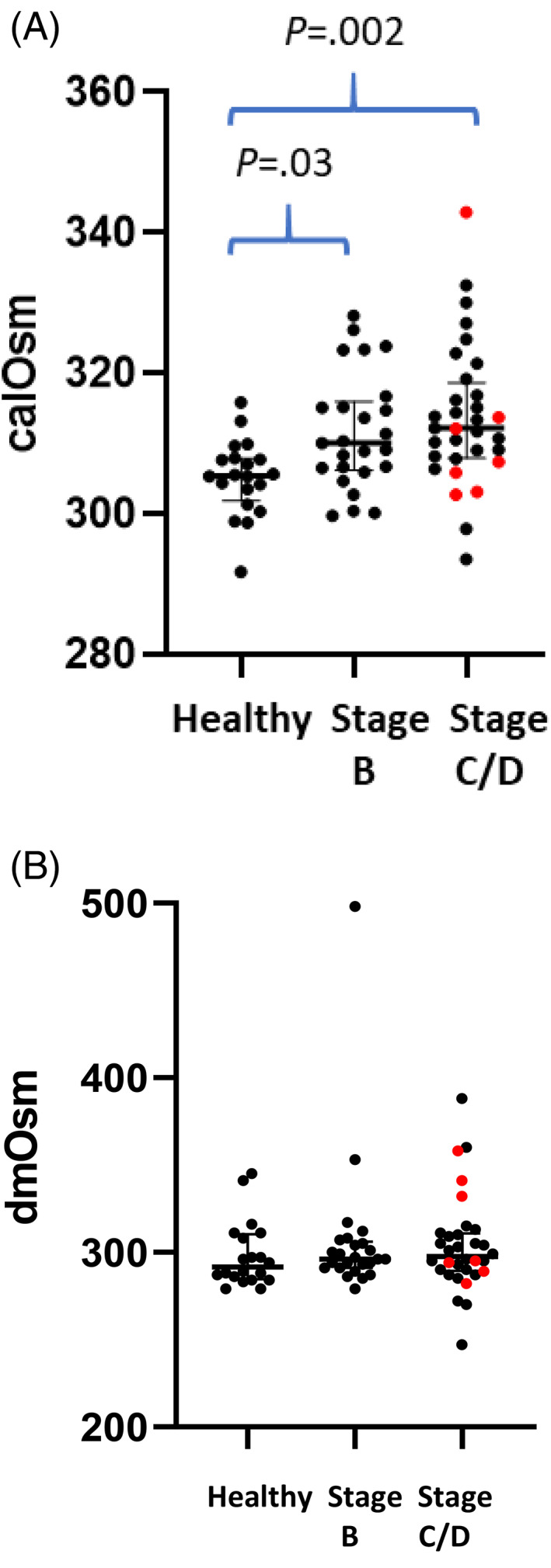
Serum osmolality is shown for healthy dogs (n = 20) and dogs of each heart disease stage (B; n = 25, C/D; n = 32). The 6 Stage D dogs are shown in red. (A) Calculated osmolality (calOsm). (B) Directly measured osmolality (dmOsm).

Calculated serum osmolality was higher in Stage B and Stage C/D dogs compared with healthy dogs (*P* = .003) but it was not different among heart disease stages. The dmOsm was not different among all groups (*P* = .48; Figure 1). The 6 Stage D dogs were not characterized by low osmolality, but 2 dogs had results <290 mOsm/kg H_2_O by direct measurement (Stage D dogs had calOsm of 343, 307, 303, 306, 314, 303 and dmOsm of 341, 289, 282, 294, 358, 295). The osmole gap (the difference between dmOsm and calOsm) was not different among groups (*P* = .34). Multiple linear regression showed no effect of heart disease stage (*P* = .69) on dmOsm. The calOsm for the dog with the highest dmOsm (498) was 323 mOsm. The glucose concentration for this dog was 87 mg/dL, BUN was 47 mg/dL, and the Na^+^ concentration was 151 mEq/L.

### 3.4 Correlations using all samples (Table 2)

**TABLE 2:** Correlations between measured variables are shown for all dogs.

Variable 1	Variable 2	*r*_s_	95% CI	*P* value
dmOsm	calOsm	0.46	0.26 to 0.63	**<.0001**
dmOsm	Na^+^	0.33	0.11 to 0.52	**.003**
dmOsm	BUN	0.37	0.15 to 0.55	**.001**
dmOsm	Glucose	0.08	−0.15 to 0.31	.48
dmOsm	Cl^−^	0.08	−0.15 to 0.30	.49
dmOsm	cCl^−^	−0.04	−0.27 to 0.19	.73
dmOsm	Amount of Cl^−^ correction	−0.33	−0.52 to −0.11	**.004**
dmOsm	HCO_3_^−^	−0.16	−0.37 to 0.08	.18
dmOsm	Anion gap	0.21	−0.02 to 0.42	.07
calOsm	Na^+^	0.79	0.68 to 0.86	**<.0001**
calOsm	BUN	0.77	0.65 to 0.85	**<.0001**
calOsm	Glucose	0.10	−0.13 to 0.32	.37
calOsm	Cl^−^	0.67	−0.17 to 0.29	.56
calOsm	cCl^−^	−0.19	−0.40 to 0.04	.10
calOsm	Amount of Cl^−^ correction	−0.78	−0.86 to −0.67	**<.0001**
calOsm	HCO_3_^−^	0.02	−0.22 to 0.25	.89
calOsm	Anion gap	0.37	0.16 to 0.56	**.001**
Cl^−^	HCO_3_^−^	−0.67	−0.78 to −0.52	**<.0001**
cCl^−^	HCO_3_^−^	−0.69	−0.79 to −0.54	**<.0001**
Amount of Cl^−^ correction	HCO_3_^−^	0.17	−0.07 to 0.38	.15
Anion gap	HCO_3_^−^	0.07	−0.16 to 0.30	.54
Cl^−^	Anion gap	−0.57	−0.71 to −0.39	**<.0001**

*Note*: Statistically significant differences at the adjusted *P* ≤ .003 are bolded. The correlation coefficient (*r*_s_) and 95% confidence interval (CI) are shown.

Abbreviations: BUN, blood urea nitrogen; Ca^+2^, calcium; calOsm, calculated osmolality; cCl^−^, corrected chloride; Cl^−^, chloride; dmOsm, direct measured osmolality; HCO_3_^−^, bicarbonate; K^+^, potassium; Na^+^, sodium; PO4^−^, phosphorus.

**FIGURE 2:**
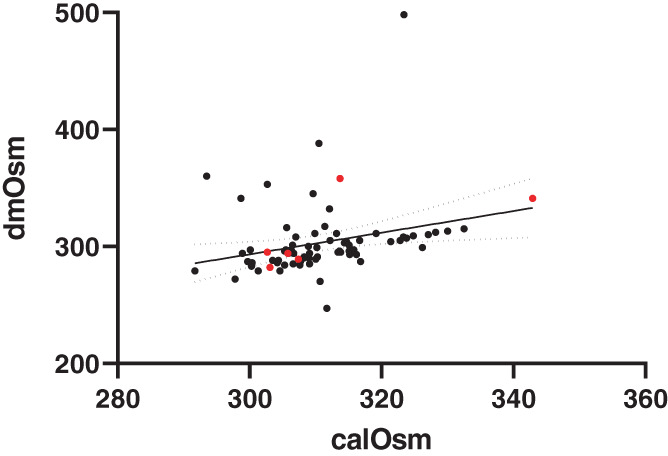
Significant and moderately positive correlation between calculated osmolality (calOsm) and directly measured osmolality (dmOsm) is shown for all 77 enrolled dogs (*P* < .0001, *r*_s_ = .46). The 6 Stage D dogs are shown in red. The best fit line and 95% confidence intervals are shown.

**FIGURE 3:**
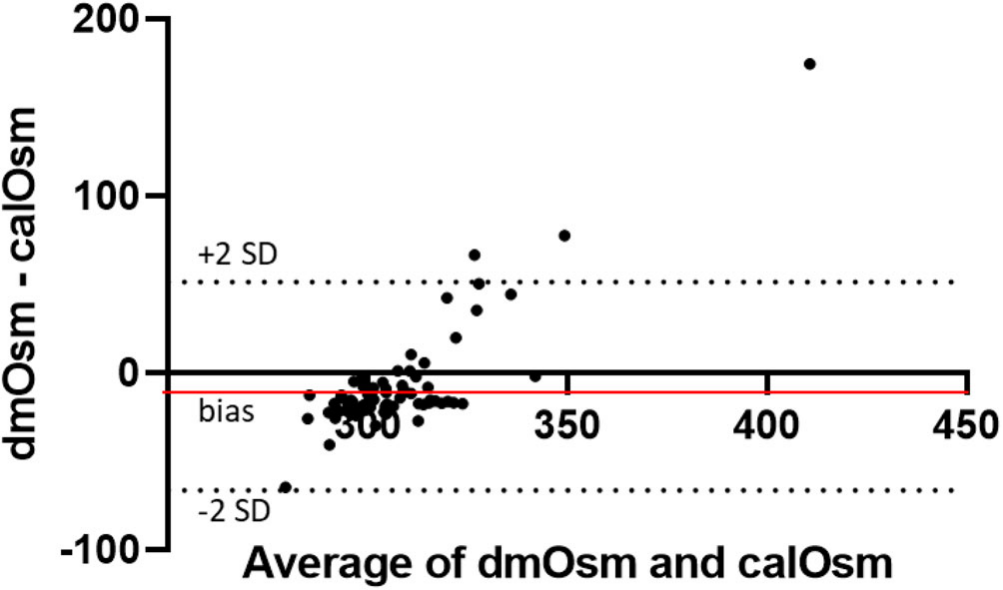
The averages of the calculated osmolality (calOsm) and directly measured osmolality (dmOsm) are plotted against the differences (dmOsm‐calOsm) using Bland‐Altman analysis for all 77 enrolled dogs. Bias (−7.5 mOsm/kg H_2_O) is shown by the red horizontal line and limits of agreement are shown as dotted horizontal lines representing ±2 standard deviations (±60 mOsm/kg H_2_O). A proportional bias is illustrated.

**FIGURE 4:**
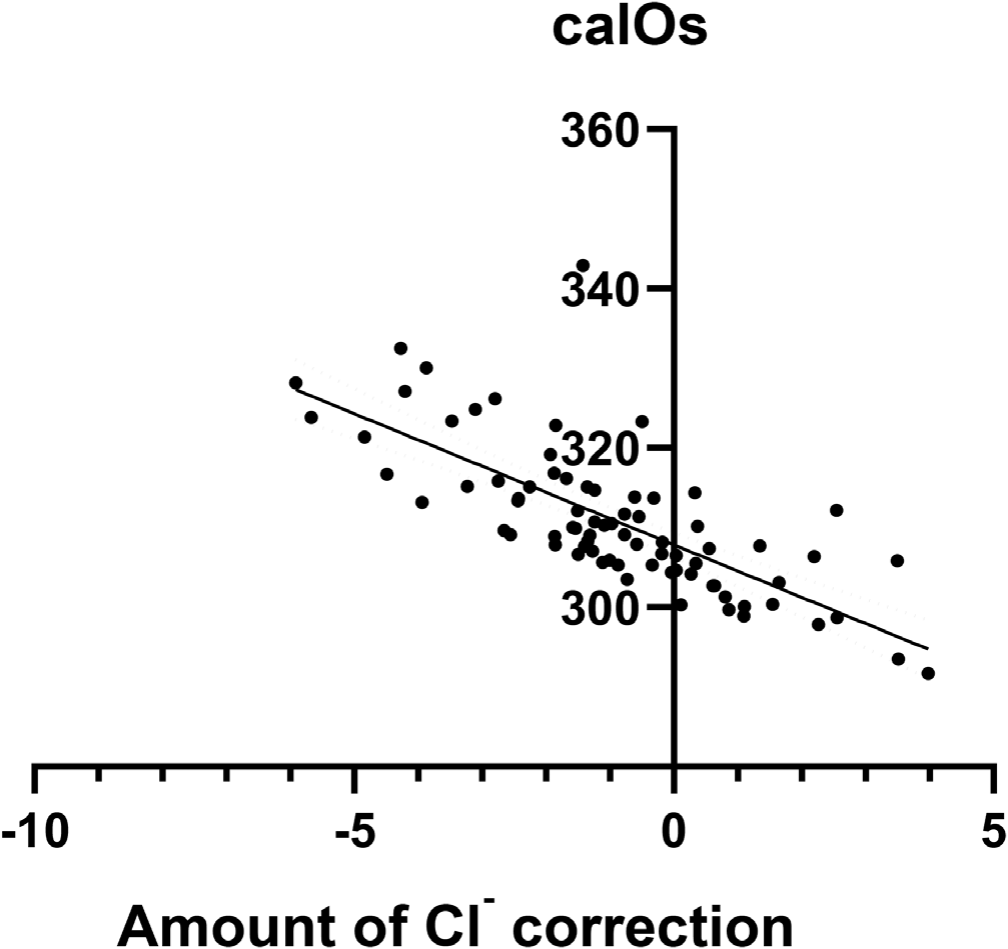
Significant and strongly negative correlation between calculated osmolality (calOsm) and amount of chloride correction is shown for all 77 enrolled dogs (*P* < .0001, *r*_s_ = −.73). The best fit line and 95% confidence intervals are shown.

The dmOsm was moderately correlated with calOsm (*r*_s_ = .46, *P* = .001; Figure 2). Bland‐Altman analysis indicated bias between the 2 methods (−7.50 mOsm/kg H_2_O) with wide limits of agreement (±60 mOsm/kg H_2_O). The bias was proportional (calOsm overestimated dmOsm at low values and underestimated dmOsm at high values (Figure 3). Osmolality by both methods was significantly and strongly negatively correlated with the amount of Cl^−^ correction (Table 2, Figure 4) and significantly and positively correlated with Na^+^ and BUN, but not with glucose.

**FIGURE 5:**
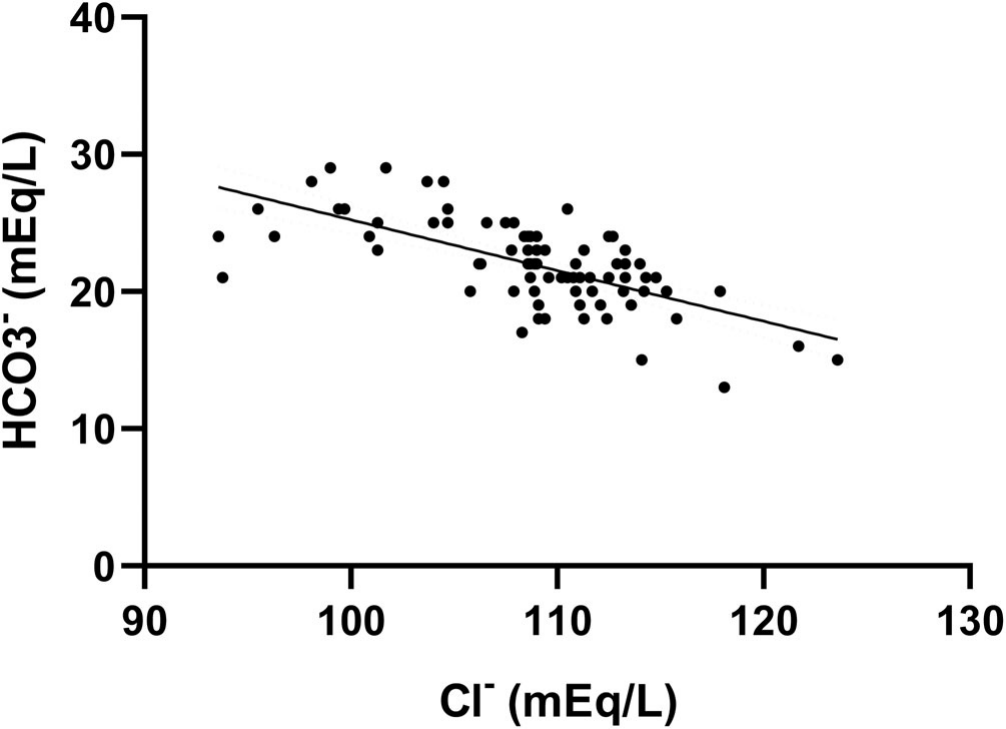
Significant and moderately negative correlation between serum bicarbonate concentration (HCO_3_^−^) and serum chloride concentration (Cl^−^) for all 77 enrolled dogs (*P* < .0001, *r*_s_ = −.67). The best fit line and 95% confidence intervals are shown.

Serum HCO_3_^−^ was moderately negatively correlated with Cl^−^ (*r*_s_ = −.67, *P* < .0001; Figure 5) and cCl^−^ (*r*_s_ = −.69, *P* < .0001). Anion gap was moderately negatively correlated with Cl^−^ (*r*_s_ = −.58, *P* < .0001).

### 3.5 Comparisons between acute and chronic Stage C dogs (Table 3)

**TABLE 3:** Comparisons between dogs with acute CHF that required hospitalization and IV furosemide and dogs with chronic CHF that did not require hospitalization and IV furosemide were made.

Variable	Acute CHF requiring IV furosemide (n = 12)	Chronic CHF not requiring IV furosemide (n = 20)	*P* value
Sex	0 MI, 5 MC, 2 FI, 5 FS	0 MI, 12 MC, 0 FI, 8 FS	.57
Stages	10 C, 2 D	16 C, 4 D	.99
Age (months)	125 (90, 156)	145 (86, 171)	.10
Weight (kg)	7.3 (2.5, 13.9)	5.5 (2.0, 10.2)	.27
Pimobendan mg/kg/day	0.67 (0.36, 1.67) (n = 10)	0.78 (0.45, 1.48) (n = 19)	.38
Furosemide mg/kg/day	5.11 (1.79, 13.39) (n = 6)	3.79 (1.33, 9.49) (n = 18)	.09
Torsemide mg/kg/day	0.36, 1.01 (n = 2)	0.26, 0.43 (n = 2)	ND
ACE‐I mg/kg/day	0.69 (0.36, 1.98) (n = 6)	0.55 (0.28, 1.28) (n = 16)	.39
Spironolactone mg/kg/day	2.01 (1.71, 3.29) (n = 5)	2.23 (0.47, 3.44) (n = 16)	.95
Glucose mg/dL	105 (62, 141)	100 (79, 133)	.65
Albumin mg/dL	3.35 (2.66, 4.23)	3.42 (2.67, 3.88)	.85
BUN mg/dL	34 (21, 59)	33 (13, 114)	.60
Creatinine mg/dL	1.16 (0.75, 2.04)	1.09 (0.44, 3.80)	.77
Na^+^ mEq/L	147.0 (141.0, 152.8)	148.2 (141.1, 152.2)	.35
K^+^ mEq/L	4.2 (3.5, 4.6)	4.6 (3.3, 5.1)	.11
Cl^−^ mEq/L	101.1 (93.6, 112.9)	107.7 (96.3, 112.5)	.01
cCl^−^ mEq/L	101.3 (95.8, 108.1)	106.0 (98.5, 109.6)	.01
Amount of Cl^−^ correction mEq/L	−0.54 (−4.84, 2.06)	−1.39 (−4.20, 3.52)	.29
HCO_3_^−^ mEq/L	25 (20, 29)	24 (17, 29)	.67
Ca^+2^ mg/dL	10.7 (9.5, 12.6)	10.7 (9.3, 12.2)	.89
PO_4_^−^ mg/dL	5.3 (3.6, 7.9)	3.7 (2.1, 6.4)	**.001**
Anion gap	25.1 (18.3, 31.0)	22.0 (18.0, 27.9)	.02
calOsm	311.2 (303.1, 332.5)	313.6 (293.5, 342.9)	.47
dmOsm	291.5 (247.0, 258.0)	302.0 (272.0, 388.0)	.08

*Note*: Median (minimum, maximum) values are shown for each variable. Unpaired *t*‐test or Mann‐Whitney test was used to compare continuous variables, while the Chi‐square test or Fisher's exact test was used to compare sex and heart disease stages between groups. Statistically significant differences at the adjusted *P* ≤ .003 are bolded.

Abbreviations: ACE‐I, angiotensin‐converting enzyme inhibitor; BUN, blood urea nitrogen; Ca^+2^, calcium; calOsm, calculated osmolality; cCl^−^, corrected chloride; Cl^−^, chloride; dmOsm, direct measured osmolality; FI, female intact; FS, female spayed; HCO_3_^−^, bicarbonate; K^+^, potassium; MI, male intact; MN, male neutered; Na^+^, sodium; ND, not done; PO_4_^−^, phosphorus.

One dog had ascites at the time of evaluation but underwent abdominocentesis and did not require hospitalization or IV furosemide. This dog was included in the chronic Stage C dogs (n = 20). All acute Stage C dogs requiring hospitalization and IV furosemide had pulmonary edema (n = 12). The median (minimum, maximum) IV furosemide dose administered to these dogs was 11.1 mg/kg (2.0, 24.5 mg/kg). Stage C dogs with acute CHF requiring hospitalization and IV furosemide had higher PO_4_^−^ (*P* = .001) compared with chronic CHF dogs.

## 4 DISCUSSION

Calculated serum osmolality was higher in dogs with heart disease (Stage B and Stage C/D) compared with healthy dogs, but there was significant overlap in group results, which limits its diagnostic value for staging. These group differences might be explained by higher BUN in dogs with heart disease compared with healthy dogs, because BUN is part of the formula used for calOsm. Interestingly, no difference was found between Stage B and Stage C/D dogs for BUN, making it unlikely that the difference is related to using diuretics to treat CHF. Additionally, no group differences were found for serum creatinine concentration. Although the BUN results for most dogs were within the reference range, the higher results, especially for Stage B compared with healthy dogs, might indicate an early decrease in renal perfusion with heart disease even in preclinical stages. This finding is consistent with a recent study that found higher urinary neutrophil gelatinase‐associated lipocalin in dogs with mitral valve disease, with results that increased with increasing heart disease severity and were unrelated to furosemide dose.^14^ Unlike calOsm findings, no differences were found among groups when osmolality was directly measured by freezing point depression. The lack of group differences for dmOsm could be related to other osmotically active substances that are directly measured and not accounted for in calculations.

We found inverse relationships between both calOsm and dmOsm with the amount of Cl^−^ correction, which was expected and supports that the amount that serum Cl^−^ changes after mathematical correction reflects water in the body, as previously proposed.^4^ Interestingly, osmolality by either method was not correlated with serum Cl^−^ concentrations or cCl^−^. Although Cl^−^ impacts osmolality, its reciprocal relationship with HCO_3_^−^ means that net negative osmoles are maintained even with hypochloremia.

Not all dogs with CHF are expected to have dilutional hypochloremia caused by non‐osmotic ADH release, but it still could be an important mechanism of diuretic resistance for some dogs with advanced disease. Indeed, our study had very few dogs with low osmolality, suggesting that water retention from non‐osmotic ADH release is uncommon in CHF. Additionally, this was not a uniform characteristic of the 6 Stage D dogs, which might mean that Stage D dogs do not have low osmolality or that the current definition of Stage D is imprecise. Several mechanisms likely contribute to poor diuretic responsiveness associated with refractory CHF, and water retention might contribute in only a small proportion of dogs. The amount of mathematical Cl^−^ correction has been proposed to reflect relative free water^4^ because this formula makes the relationship between Na^+^ and Cl^−^ readily apparent in a clinical scenario where the expected 1.3:1 ratio might be disrupted because of diuretics or neurohormonal stimulation. The formula corrects for dilution or concentration of serum Na^+^, which might not be readily recognized by looking at the uncorrected results alone. Our data supported this conclusion, especially when Na^+^ concentrations were within the reference range. Therefore, dogs with advanced heart disease that have clinically relevant increases in serum Cl^−^ after mathematical correction should have lower calOsm, consistent with non‐osmotic ADH, that causes relative free water retention. This relationship was supported by our data. The singular and unvalidated distinction of Stage D from Stage C using loop diuretic dosage could impact studies attempting to investigate distinguishing characteristics of the stage because furosemide dosage is subject to factors other than poor diuretic responsiveness, including clinician preferences for dosing. Therefore, the current Stage D definition is a guide and is not considered a gold standard for identifying refractory CHF, which implies diuretic resistance. We propose incorporating other factors, such as low urine Na^+^ ^15^ and the amount of upward Cl^−^ correction, into the definition of Stage D CHF dogs.

The lack of a relationship between Cl^−^ and calOsm is explained by osmolality formulas which do not account for this anion, but rather assume a 1:1 relationship between cations and anions, with Na^+^ being the major cation used in formulas. It is unnecessary to include Cl^−^ in calculations because of the reciprocal relationship of Cl^−^ and HCO_3_^−^, which are physiologically balanced to maintain serum osmolality in the animal. Therefore, reciprocal changes in Cl^−^ and HCO_3_^−^ that occur to maintain normal osmolality when either of these electrolytes is disrupted by, for example, loop diuretics or acid‐base disturbances, would affect their individual relationships with Osm, precluding close correlations. Our study affirmed this inverse relationship between Cl^−^ and HCO_3_^−^. This reasoning also explains why the calOsm was significantly and strongly positively correlated with both Na^+^ and BUN, because both solutes are part of calOsm equations. However, unexpectedly and as previously shown in a study of people, neither calOsm nor dmOsm correlated with glucose, raising the question of whether it is necessary to include glucose in osmolality equations for non‐diabetic patients.^16^

One dog in our study had very high dmOsm (498 mOsm/kg) and only mildly high calOsm. The BUN in this dog was mildly increased (47 mg/dL), but other reasons for the very high dmOsm were not apparent, and the sample was handled in the same manner as the other samples without apparent measurement error. Assuming that dmOsm is the gold standard, our results, especially the poor method agreement with proportional bias, raise questions about the validity of calculated osmolality equations in dogs with heart disease.

The calOsm and dmOsm were moderately correlated, but a negative osmole gap was observed, indicating that calOsm overestimated dmOsm. The negative bias obtained when the methods were compared using Bland‐Altman analysis also demonstrated the negative osmole gap. Still, the degree and direction of the bias were discordant at high and low osmolality. This proportional bias and the wide limits of agreement indicate that the 2 methods for determining osmolality cannot be used interchangeably. A previous study also reported a negative osmole gap using the freezing point depression method to analyze serum samples.^9^ These samples were stored at 7°C for 7 days and then stored at −80°C until the time of analysis, which occurred within 30 days of sample collection.^9^ Assuming dmOsm is the gold standard, our results, in combination with the previous study, indicate that although the 2 methods correlate with each other, there are likely factors other than Na^+^, BUN, and glucose that influence serum osmolality and should be considered in formulas designed to calculate serum osmolality.^16^ A negative osmole gap also has been reported for dmOsm obtained using vapor pressure osmometry, but explanations were not explored in that study.^16^ Our study utilized freezing point depression, which is considered more accurate than vapor pressure over an analytical range of 0 to 2000 mmol/kg with refrigerated samples.^17^ Although this study only used refrigerated samples (4°C), 1 study in dogs found a small and probably clinically irrelevant effect of short‐term freezing on dmOsm, possibly associated with altered concentrations of other solutes, protein degradation, or protein aggregation, although the processes are poorly understood.^10^ Short‐term storage at −18°C for some samples might have affected our results, but the minor effect in previous studies and the lack of influence of temperature on our results when evaluated statistically indicate that storage time likely had minimal impact on the study findings.

Clinical variables and osmolality for Stage C/D dogs were compared on the basis of whether the dogs were presented for acute CHF decompensation or were stable and without active evidence of congestion at the time of evaluation. Only serum PO_4_^−^ was significantly higher after controlling for multiple comparison testing, although lower Cl^−^ and cCl^−^ approached significance. Higher PO_4_^−^ in dogs presenting with acute CHF might be explained by decreased renal elimination from decreased renal perfusion, but other mechanisms related to IV loop diuretics are also possible.

As expected, based on renal physiology principles, serum HCO_3_^−^ was negatively correlated with serum Cl^−^ and cCl^−^ but not the amount of Cl^−^ correction. This disconnect highlights the different reasons for hypochloremia in CHF: depletion, which is a true deficit and inversely related to increased serum HCO_3_^−^, vs dilutional, which is inversely related to upward mathematical correction of Cl^−^ because of water retention. The inverse relationship between HCO_3_^−^ and Cl^−^ previously has been reported in dogs without heart disease,^18^ and our results were similar in this sample of dogs with heart disease, some of which were receiving diuretics, which can affect the relationship between other electrolytes and HCO_3_^−^.

Limitations of our study include small sample size for Stage D dogs and the unvalidated definition of Stage D, precluding separate analysis from Stage C dogs. Although we had a control group of healthy dogs, they were not well matched for age and weight, with healthy dogs being younger and heavier. These differences might have impacted group comparisons. Another potential limitation might be the manual calibration required for the freezing point depression osmometer used in our study, which could have introduced human error.^17^ An additional limitation is that samples were held at 2 different temperatures before direct osmometry. The freezing point depression method does not eliminate cryoscopic factors such as high viscosity and insoluble particles arising from freezing and thawing the samples. However, we did not find a significant effect of freezing on dmOsm results.^17^

In conclusion, our results support using the amount of Cl^−^ correction as an inverse indicator of calOsm and possibly water retention in dogs with heart disease. We found that dogs with heart disease had higher calOsm than healthy dogs, which appears to be associated with differences in BUN between these groups of dogs. We did not find that dogs with CHF as a whole had lower serum osmolality than dogs without CHF, but some individual dogs had low osmolality results and might have had non‐osmotic stimulation of ADH release causing water retention. A larger study of dogs with documented diuretic resistance would be required to test this possibility further. Lastly, the lack of method agreement between calOsm and dmOsm, and previous studies demonstrating that a single equation might not be applicable for all clinical patient populations indicates a need to develop a calOsm equation that predicts dmOsm in dogs with heart disease over a range of values.^16^, ^19^

## CONFLICT OF INTEREST DECLARATION

Authors declare no conflict of interest.

## OFF‐LABEL ANTIMICROBIAL DECLARATION

Authors declare no off‐label use of antimicrobials.

## INSTITUTIONAL ANIMAL CARE AND USE COMMITTEE (IACUC) OR OTHER APPROVAL DECLARATION

Approved by the IACUC of the University of Florida for blood collection for this study (202120518).

## HUMAN ETHICS APPROVAL DECLARATION

Authors declare human ethics approval was not needed for this study.

## Supporting information


**Table S1.** Comparisons between Stage B1 and Stage B2 dogs. Median (minimum, maximum) values are shown for each variable. ACE‐I, angiotensin‐converting enzyme inhibitor; BUN, blood urea nitrogen; Ca^+2^, calcium; calOsm, calculated osmolality; cCl^−^, corrected chloride; Cl^−^, chloride; dmOsm, direct measured osmolality; FI, female intact; FS, female spayed; HCO_3_^−^, bicarbonate; K^+^, potassium; MI, male intact; MN, male neutered; Na^+^, sodium; ND, not done; PO_4_^−^, phosphorous.
