# Supplementary material for: Use of serum osmolality to identify heart disease stage in dogs and relationship to mathematical chloride correction
Source: J Vet Intern Med. 2023 Sep 13;37(6):2011–20. doi: 10.1111/jvim.16863 (PMC10658530; doi:10.1111/jvim.16863)
Supplement: Supplementary file 1 — Table S1. Comparisons between Stage B1 and Stage B2 dogs. Median (minimum, maximum) values are shown for each variable. ACE‐I, angiotensin‐converting enzyme inhibitor; BUN, blood urea nitrogen; Ca+2, calcium; calOsm, calculated osmolality; cCl−, corrected chloride; Cl−, chloride; dmOsm, direct measured osmolality; FI, female intact; FS, female spayed; HCO3 −, bicarbonate; K+, potassium; MI, male intact; MN, male neutered; Na+, sodium; ND, not done; PO4 −, phosphorous. [file JVIM-37-2011-s001.pdf]

Supplemental Table 1. Comparisons between Stage B1 and Stage B2 dogs. Median (minimum, maximum) values are shown for each variable. MI; male intact, MN; male neutered, FI; female intact, FS; female spayed, ACE-I; angiotensin-converting enzyme inhibitor, BUN; blood urea nitrogen, Na^+; sodium, K^+; potassium, Cl^-; chloride, cCl^-; corrected chloride, $HCO3^-$; bicarbonate, Ca^{+2}; calcium, $PO4^-$; phosphorous, calOsm; calculated osmolality, dmOsm; direct measured osmolality, ND; not done.

**Variable**	**B1 (n=10)**	**B2 (n=15)**	***P* value**
**Sex**	1 MI, 5 MC, 0 FI, 4 FS	1 MI, 6 MC, 0 FI, 8 FS	.51
**Age (months)**	131 (57, 181)	146 (96, 190)	.4
**Weight (kg)**	6.4 (3.5, 30.7)	7.2 (2.4, 15.8)	.88
**Pimobendan mg/kg/day**	n=0	0.62, (0.47, 0.96) (n=15)	ND
**IV furosemide if hospitalized**	n=0	n=0	ND
**Furosemide mg/kg/day**	1.7 (n=1)	n=0	ND
**Torsemide mg/kg/day**	n=0	n=0	ND
**ACE-I mg/kg/day**	1.16 (0.86, 1.45)	0.52 (0.38, 0.95) (n=3)	ND
**Spironolactone mg/kg/day**	n=0	3.5 (3.16, 3.85) (n=2)	ND
**Glucose mg/dL**	95 (43, 122)	93 (61, 117)	.79
**Albumin mg/dL**	3.27 (2.82, 4.16)	3.04 (2.22, 3.82)	.29
**BUN mg/dL**	23 (15, 58)	24 (13, 70)	.93
**Creatinine mg/dL**	1.02 (0.71, 1.23)	0.80 (0.55, 1.99)	.91
**Na^+ mEq/L**	147.7 (144.2, 153.6)	148.4 (145.1, 153.4)	.43
**K^+ mEq/L**	4.5 (4.0, 5.3)	4.5 (3.8, 5.5)	.74
**Cl^- mEq/L**	110.7 (108.9, 123.6)	110.2 (106.6, 121.7)	.35
**cCl^- mEq/L**	110.4 (106.2, 119.2)	109.3 (106.1, 116.0)	.18

**Amount of Cl^- correction mEq/L**	-1.04 (-5.91, 1.55)	-1.58 (-5.62, 0.86)	.45
**$HCO3^-$ mEq/L**	21 (13, 26)	21 (16, 25)	.16
**Ca^{+2} mg/dL**	10.2 (9.4, 10.8)	10.7 (9.5, 11.3)	.09
**$PO4^-$ mg/dL**	3.3 (2.3, 4.0)	3.6 (2.7, 5.3)	.14
**Anion gap**	20.1 (14.8, 25.8)	20.6 (17.3, 23.5)	.81
**calOsm**	307.9 (300.1, 328.2)	311.4 (299.7, 323.8)	.62
**dmOsm**	294.0 (279.0, 312.0)	300.0 (285.0, 498.0)	.25
